# A comprehensive analysis of m6A/m7G/m5C/m1A-related gene expression and immune infiltration in liver ischemia–reperfusion injury by integrating bioinformatics and machine learning algorithms

**DOI:** 10.1186/s40001-024-01928-y

**Published:** 2024-06-13

**Authors:** Zhanzhi Meng, Xinglong Li, Shounan Lu, Yongliang Hua, Bing Yin, Baolin Qian, Zhongyu Li, Yongzhi Zhou, Irina Sergeeva, Yao Fu, Yong Ma

**Affiliations:** 1 Department of Minimally Invasive Hepatic Surgery, The First Affiliated Hospital of Harbin Medical University, Harbin, 150001 China; 2 Key Laboratory of Hepatosplenic Surgery, Ministry of Education, The First Affiliated Hospital of Harbin Medical University, Harbin, China; 3 Department of Pediatric Surgery, The First Affiliated Hospital of Harbin Medical University, Harbin, China; 4 Department of Ultrasound, The First Affiliated Hospital of Harbin Medical University, Harbin, China

**Keywords:** Liver ischemia–reperfusion injury, Methylation modification, Immune infiltration, Machine learning, Bioinformatics

## Abstract

**Background:**

Liver ischemia–reperfusion injury (LIRI) is closely associated with immune infiltration, which commonly occurs after liver surgery, especially liver transplantation. Therefore, it is crucial to identify the genes responsible for LIRI and develop effective therapeutic strategies that target immune response. Methylation modifications in mRNA play various crucial roles in different diseases. This study aimed to identify potential methylation-related markers in patients with LIRI and evaluate the corresponding immune infiltration.

**Methods:**

Two Gene Expression Omnibus datasets containing human liver transplantation data (GSE12720 and GSE151648) were downloaded for integrated analysis. Gene Ontology and Kyoto Encyclopedia of Genes and Genomes pathway enrichment analyses were conducted to investigate the functional enrichment of differentially expressed genes (DEGs). Differentially expressed methylation-related genes (DEMRGs) were identified by overlapping DEG sets and 65 genes related to *N*6-methyladenosine (m6A), 7-methylguanine (m7G), 5-methylcytosine (m5C), and *N*1-methyladenosine (m1A). To evaluate the relationship between DEMRGs, a protein–protein interaction (PPI) network was utilized. The core DEMRGs were screened using three machine learning algorithms: least absolute shrinkage and selection operator, random forest, and support vector machine-recursive feature elimination. After verifying the diagnostic efficacy using the receiver operating characteristic curve, we validated the expression of the core DEMRGs in clinical samples and performed relative cell biology experiments. Additionally, the immune status of LIRI was comprehensively assessed using the single sample gene set enrichment analysis algorithm. The upstream microRNA and transcription factors of the core DEMRGs were also predicted.

**Results:**

In total, 2165 upregulated and 3191 downregulated DEGs were identified, mainly enriched in LIRI-related pathways. The intersection of DEGs and methylation-related genes yielded 28 DEMRGs, showing high interaction in the PPI network. Additionally, the core DEMRGs *YTHDC1*, *METTL3*, *WTAP*, and *NUDT3* demonstrated satisfactory diagnostic efficacy and significant differential expression and corresponding function based on cell biology experiments. Furthermore, immune infiltration analyses indicated that several immune cells correlated with all core DEMRGs in the LIRI process to varying extents.

**Conclusions:**

We identified core DEMRGs (*YTHDC1*, *METTL3*, *WTAP*, and *NUDT3*) associated with immune infiltration in LIRI through bioinformatics and validated them experimentally. This study may provide potential methylation-related gene targets for LIRI immunotherapy.

**Supplementary Information:**

The online version contains supplementary material available at 10.1186/s40001-024-01928-y.

## Background

Ischemia–reperfusion injury (IRI), a pathophysiological process, involves two stages: reduction or interruption of blood supply and subsequent reperfusion and reoxygenation of the target organs [1]. Several organs, including the liver, are known to be at high risk of developing IRI [2–5]. Liver IRI (LIRI) is an unavoidable condition that occurs during liver transplantation or resection (particularly when using the Pringle maneuver) [6, 7]. During the initial stage, the ischemic microenvironment exposes liver cells to hypoxia, ion disorder, and ATP depletion, leading to the accumulation of reactive oxygen species, calcium overload, cell injury, or even death [8]. Instead of restoration after blood reperfusion, the liver cells suffer from damage and various forms of death due to the release of proinflammatory cytokines by immunocytes, mitochondrial dysfunction, and subsequent activation of multiple inflammatory cascades [9]. The intricate network of pathophysiological mechanisms makes it challenging to search for more effective therapeutic targets that can potentially improve LIRI. Thus, it is necessary to identify novel target genes to treat LIRI.

In 2005, Professor Land stated that a proinflammatory innate immune-dominated response may induce an adaptive immune response in IRI [10]. The LIRI-induced immune cascade is divided into two distinct stages. Initially, Kupffer cells are activated by sensing the damage-associated molecular patterns, which are expressed on cells experiencing stress or damage, and subsequently secrete chemokines and cytokines. During the second stage, as circulating monocytes, neutrophils, and T cells are recruited for assistance, liver cells are further harmed [11]. As the immunologic process plays an indispensable role in LIRI, further exploration of the regulatory mechanism of immune cell activation in LIRI can offer potential targets for disease prediction and therapeutic intervention.

In 1942, Dr. Waddington suggested a link between genotype and phenotype for the first time and proposed the name “epigenotype,” indicating the expression of genetic material throughout the life process in terms of the whole organism [12]. After the discovery of pseudouridine (Ψ), post-transcriptional RNA modification was first revealed, and researchers started exploring gene regulation at the RNA level [13]. Since then, the terms “RNA epigenetics” and “epitranscriptomics” have been successively coined [14, 15]. From the perspective of epigenetics, however, the intricate interplay of DNA methylation, histone acetylation, and RNA modification contributes to the development of IRI to a varying extent [16, 17]. Unlike DNA or histones, the types of RNA modifications are greater in number, with > 170 modifications identified to date, including *N*6-methyladenosine (m6A), 7-methylguanine (m7G), 5-methylcytosine (m5C), and *N*1-methyladenosine (m1A) [18]. Previous studies have indicated that RNA methylation affects the tumor immune microenvironment in hepatocellular carcinoma as well as the activities of various immune cells [19, 20]. It has been demonstrated that m6A, the most prevalent form of methylation modification, plays an important role in the progression of IRI [17]. Previous studies have suggested that kidney-specific knockout of methyltransferase-like 3 (METTL3) may alleviate IRI-induced renal dysfunction, renal injury, and renal inflammation by inhibiting TAB3 m6A modification through insulin-like growth factor 2 mRNA binding protein 2 [21]. Moreover, the demethylase fat mass and obesity-associated protein (FTO) reduce the stability of cGAS mRNA during the progression of cerebral IRI, thereby alleviating brain inflammation by inhibiting the STING/NF-κB axis [22]. Recently, m7G modification was found to be associated with various diseases, including hepatocellular carcinoma, postischemic angiogenesis, heart failure, and cardiac fibrosis due to myocardial ischemia [23–25]. Similarly, the role of m5C in cells is being gradually recognized, with ALKBH1 being identified as essential for mitochondrial function, thereby influencing atherosclerosis [26]. In addition, there is evidence that the abundance of m1A significantly reduces after cerebral ischemia and influences biological regulation following stroke [27]. However, studies on whether these RNA methylation modifications play crucial roles in LIRI and how the relevant immune cells participate in biological processes remain limited, and further studies are urgently warranted.

To address these research gaps, our study leveraged two Gene Expression Omnibus (GEO) datasets containing gene expression data from human liver transplantation samples, representing the states of ischemia and reperfusion. Furthermore, we identified significantly differentially expressed genes (DEGs) and used previously identified m6A/m7G/m5C/m1A regulators, among which differentially expressed methylation-related genes (DEMRGs) were identified. Machine learning algorithms were employed to identify characteristic core DEMRGs, which were validated through real time-quantitative polymerase chain reaction (RT-qPCR) using clinical liver transplant specimens. We also performed cytological experiments to detect alterations in methylation levels following LIRI. Subsequently, functional validation was performed after modulating the expression levels of specific genes in cells. Additionally, the association between DEMRGs and infiltration of immune cells was examined to gain a more profound insight into the molecular immunological mechanisms related to methylation in the progression of LIRI.

## Methods

### Data acquisition and processing

Data acquisition was performed using the GEO database (https://www.ncbi.nlm.nih.gov/geo/), a public repository of high-throughput gene expression data [28]. We identified relevant GEO datasets based on the following inclusion criteria. Organism: *Homo sapiens*; experiment type: expression profiling via array or high-throughput sequencing; procedure: orthotopic liver transplantation; and sample types: pre- and post-transplant liver tissues. We then used the search terms “liver transplantation” as the MeSH term and “*Homo sapiens*” as the organism to detect prospective GEO datasets. We performed additional screening of datasets based on patient inclusion criteria, donor status, and specimen acquisition methods. Ultimately, two GEO datasets, namely, GSE12720 and GSE151648, were deemed eligible and then included in our study (Additional file 1: Table S1). To conduct additional analyses, we used clinical information from both datasets as covariates and employed the ComBat function from the ‘sva’ R package to eliminate batch effects between the two datasets [29]. To homogenize gene expression profiles, the “preprocessCore” package was used.

The regulators of m6A, m7G, m5C, and m1A modifications were obtained from prior research [19, 30].

### Identification of DEGs and DEMRGs

The “limma” package in R software was utilized to detect DEGs. Adjusted *P*-value of < 0.05 was used as a criterion to identify significantly different expressions between pre-transplant and post-transplant groups. Furthermore, the packages “pheatmap” and “ggplot2” were employed to construct volcano and heatmap plots of DEGs. To identify DEMRGs, we decided to overlap methylation-related gene sets with upregulated and downregulated DEG sets separately.

### Functional enrichment analyses

Functional enrichment analyses of Gene Ontology (GO), including three categories—biological process (BP), cellular component (CC), and molecular function (MF)—and Kyoto Encyclopedia of Genes and Genomes (KEGG) were conducted using the R package “clusterProfiler” [31].

### Identification and validation of core DEMRGs

In this study, DEMRGs that play a crucial role through methylation modification during the LIRI process were considered core DEMRGs. These genes were screened using three machine learning algorithms: least absolute shrinkage and selection operator (LASSO) logistic regression [32], random forests (RF) [33], and support vector machine-recursive feature elimination (SVM-RFE) [34]. This study performed LASSO logistic regression analysis using the R package “glmnet” [35]. The random forest method was applied using the R package “randomForest.” The SVM-RFE method was performed utilizing the R software package “kernlab.” To assess diagnostic efficacy, we constructed receiver operating characteristic (ROC) curves for all core DEMRGs and calculated the area under the ROC curve (AUC) values using the R package “pROC.”

### Immune infiltration analysis

To examine the extent of infiltration and differences in gene expression among 23 immune cell types in pre-transplant and post-transplant groups, we use the single sample gene set enrichment analysis (ssGSEA) algorithm based on the “GSVA” package. A significant alteration in LIRI progression was observed for certain types of immune cells when the *P*-values were < 0.05. The correlation between core DEMRGs and immune cells was analyzed using “ggplot2” in R, and only results with *P*-values of < 0.05 were displayed in the lollipop diagrams.

### Gene set enrichment analysis

We performed a correlation analysis to examine the relationship between the core DEMRGs and all genes in the GEO datasets. Heatmaps displayed the top 50 genes that exhibited a positive correlation with the core DEMRGs. Based on the findings of correlation analysis, we further conducted a gene set enrichment analysis (GSEA) using the REACTOME pathway browser (https://reactome.org) [36]. The mountain map was used to present the top 20 pathways associated with each core DEMRG. Pathways with a *P*-value of < 0.05 were deemed statistically significant.

### Construction of protein–protein interaction (PPI) network and regulatory network of microRNAs and transcription factors

We uploaded DEMRGs onto the Search Tool for the Retrieval of Interacting Genes (STRING) online database (http://string-db.org) to construct a related PPI network [37]. To further explore gene regulation mechanisms, we predicted upstream microRNAs (miRNAs) and transcription factors of the core DEMRGs by utilizing the regulatory network repository of transcription factor and microRNA-mediated gene regulations (RegNetwork) [38]. The network was input into Cytoscape software for visualization.

### Clinical sample collection

We collected 10 pairs of liver tissue samples from 10 patients who underwent liver transplantation at the First Affiliated Hospital of Harbin Medical University. Each pair consisted of pre-transplant and post-transplant samples. The pre-transplant sample was obtained from the donor’s liver at the beginning of surgery, representing the non-LIRI state. The sample from the post-transplant group, taken 2 h after allograft revascularization, represents the LIRI state. The collection protocols aligned with those of GSE151648 data methods. A professional pathologist was asked to assess whether the samples were usable in a blinded fashion. Written informed consent was obtained from all patients. This study was conducted in line with the guidelines of the Declaration of Helsinki and was approved by the ethics committee of the First Affiliated Hospital of Harbin Medical University.

### Animal

Eight-week-old male C57BL/6 mice weighing 19–23 g were obtained from Vital River (Beijing, China) and housed under standard conditions (temperature: 23 °C ± 2 °C, 12-h light/dark cycle) before surgery. All animal procedures were approved by the Ethics Committees of Harbin Medical University and conducted in accordance with the NRC’s Guide for the Care and Use of Laboratory Animals.

### Isolation of primary mouse hepatocytes

Primary mouse hepatocytes were isolated following established procedures [39]. Briefly, mice were fully anesthetized, and their abdominal cavities were opened. Sequential perfusion of Hanks Balanced Salt Solutions (HBSS) and 0.05% IV collagenase solutions was performed through the portal vein. Liver tissues were dissected and incubated in 0.05% IV collagenase solution for 20 min. After digestion, a cell suspension was obtained through filtration (70 μm). The hepatocytes were collected by rinsing with PBS, followed by centrifugation.

### Small interfering RNA (siRNA)

siRNAs, which were custom-designed and synthesized by Hanheng Biotechnology (Shanghai, China), were employed to silence gene expression. The sequences used are listed in Additional file 2: Table S2.

### siRNA transfection

The siRNAs exhibiting the highest inhibition rates against the target genes were selected for subsequent functional experiments. Two experimental groups were established: the siRNA group, in which the cells were transfected with specific siRNAs, and the negative control (NC) group, in which the cells were transfected with nontargeting NC siRNAs. Briefly, 24 h before transfection, primary mouse hepatocytes were seeded into 6-well plates at a density of 2 × 10^5^ cells per well to ensure 30–50% confluency at the time of transfection. In the siRNA group, the cells were transfected with the specific siRNA at a concentration of 100 nmol/L using Lipofectamine 3000 (Invitrogen, USA). Similarly, in the NC group, the cells were transfected with NC siRNA at the same concentration. The transfections were performed in triplicate at each time point. At various time points following the initiation of transfection, the cells were harvested for subsequent assays.

### Hypoxia/reoxygenation (H/R) model construction

Hepatocytes in a good state were screened and cultured overnight, and the medium was replaced with glucose- and serum-free DMEM. Subsequently, the cells were transferred to a chamber (Biospherix, Lacona, NY, USA) under an atmosphere of 1% O_2_, 5% CO_2_, and 94% N_2_ for 6 h. Then, the medium was replaced with DMEM supplemented with 10% serum, and the cells were incubated under normoxic conditions (95% air and 5% CO_2_) for an additional 6 h. The medium in the control group was replaced with DMEM supplemented with 10% serum, and the cells were continuously cultured under normoxic conditions.

### Cell viability assay

Cell viability was assessed using a CCK-8 kit obtained from Dongren Chemical Technology (Shanghai, China). Following transfection with specific siRNAs, the cells were seeded into 96-well plates at a density of 5 × 10^3^ cells per well in triplicate for each experimental group and subjected to H/R treatment. After a 2-h incubation at 37 °C in an incubator protected from light, the optical density was measured.

### Dot blot

RNA was extracted from primary mouse hepatocytes subjected to H/R stimulation and dotted onto a nitrocellulose (NC) membrane. The RNA was cross-linked with the membrane via ultraviolet irradiation after the membrane was dried naturally in the air. Methylene blue stain was used as a loading control. The membrane was washed with TBST, blocked with 5% BSA in TBST 1 h at room temperature, and then incubated at 4 °C overnight with m6A-specific antibody (Proteintech, 68055-1-Ig, 1:1000) or m7G-specific antibody (Proteintech, 68302-1-Ig, 1:2500). The following day, the membrane was incubated with a fluorescent secondary antibody for 2 h. The Odyssey Infrared imaging system was used for imaging.

### RT-qPCR

Total RNA was extracted from the liver allograft biopsy sample using a commercial TRIzol reagent (AxyPrep Multisource Total RNA Miniprep Kit, Axygen®). RNA was used to synthesize complementary DNA (cDNA) using a cDNA synthesis kit (ReverTra Ace™ qPCR RT Master Mix, TOYOBO Inc., Japan). The primer sequences were designed and created by Generalbiol (Chuzhou, China) (Additional file 3: Table S3). RT-qPCR was performed using a ready-to-use master mix (FastStart™ Universal SYBR® Green Master, Rox), and data analysis was performed via the 2^−ΔΔCt^ method using GAPDH as a reference gene.

### Statistical analysis

Bioinformatics analyses and statistical tests were performed using R software version 4.2.2. The differences in individual genes between the two groups were assessed using the Student’s *t*-test. Pearson’s correlation test was used to evaluate relationships between variables. Adjusted *P*-values of < 0.05 were considered to indicate statistical significance.

## Results

### Identification of DEGs related to LIRI

**Fig. 1:**
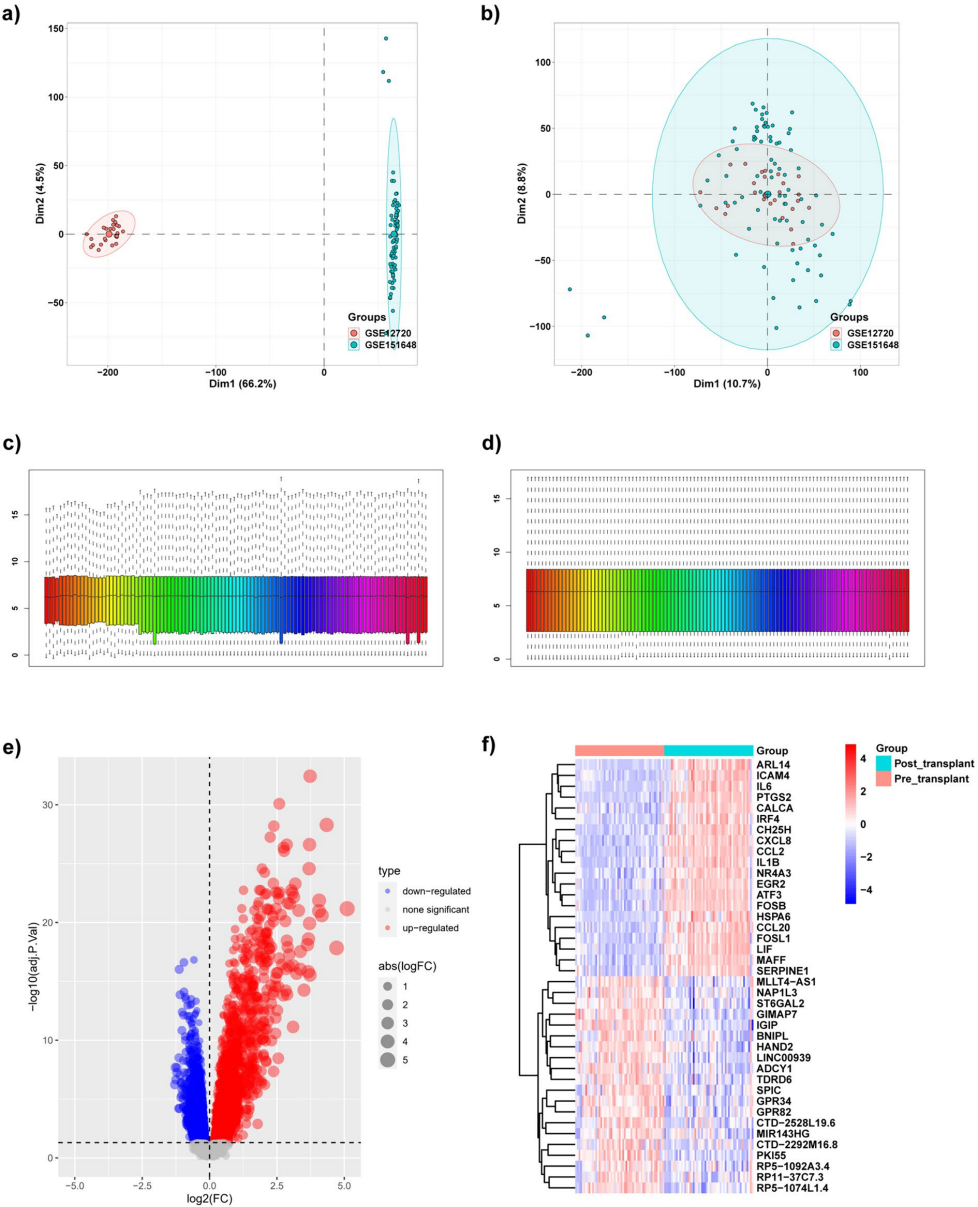
Identification of DEGs. **a** PCA cluster plot of GSE12720 and GSE151648 before batch effect removal and correction. **b** The PCA cluster plot showed that the batch effect has been removed. **c** The gene expression box chart before normalization. **d** The gene expression box chart displayed normalized gene arrays. **e** Volcano plot of DEGs between pre-transplant and post-transplant groups. **f** Heatmap for the top 20 upregulated and down-regulated DEGs between pre-transplant and post-transplant groups. Red: up-regulation, Blue: down-regulation. *DEGs* differentially expressed genes, *FC* foldchange

In this study, two eligible datasets, namely, GSE12720 and GSE151648, were selected. We merged gene expression profiles from these two datasets and removed the batch effect using the “sva” package. The PCA cluster plots were displayed using the “FactoMineR” package and “factoextra” package, which explicitly showed that the batch effect was efficiently removed (Fig. 1a, b). We obtained 19,426 genes and 106 samples after removing the batch effect. Expression data were normalized using the “preprocessCore” package (Fig. 1c, d). After setting the parameter of adjusted *P*-value < 0.05, DEGs were identified using the “limma” package. Volcano plot and heatmap displayed genes that were upregulated and downregulated following LIRI (Fig. 1e, f).

### Biological processes and pathway enrichment analysis of DEGs

**Fig. 2:**
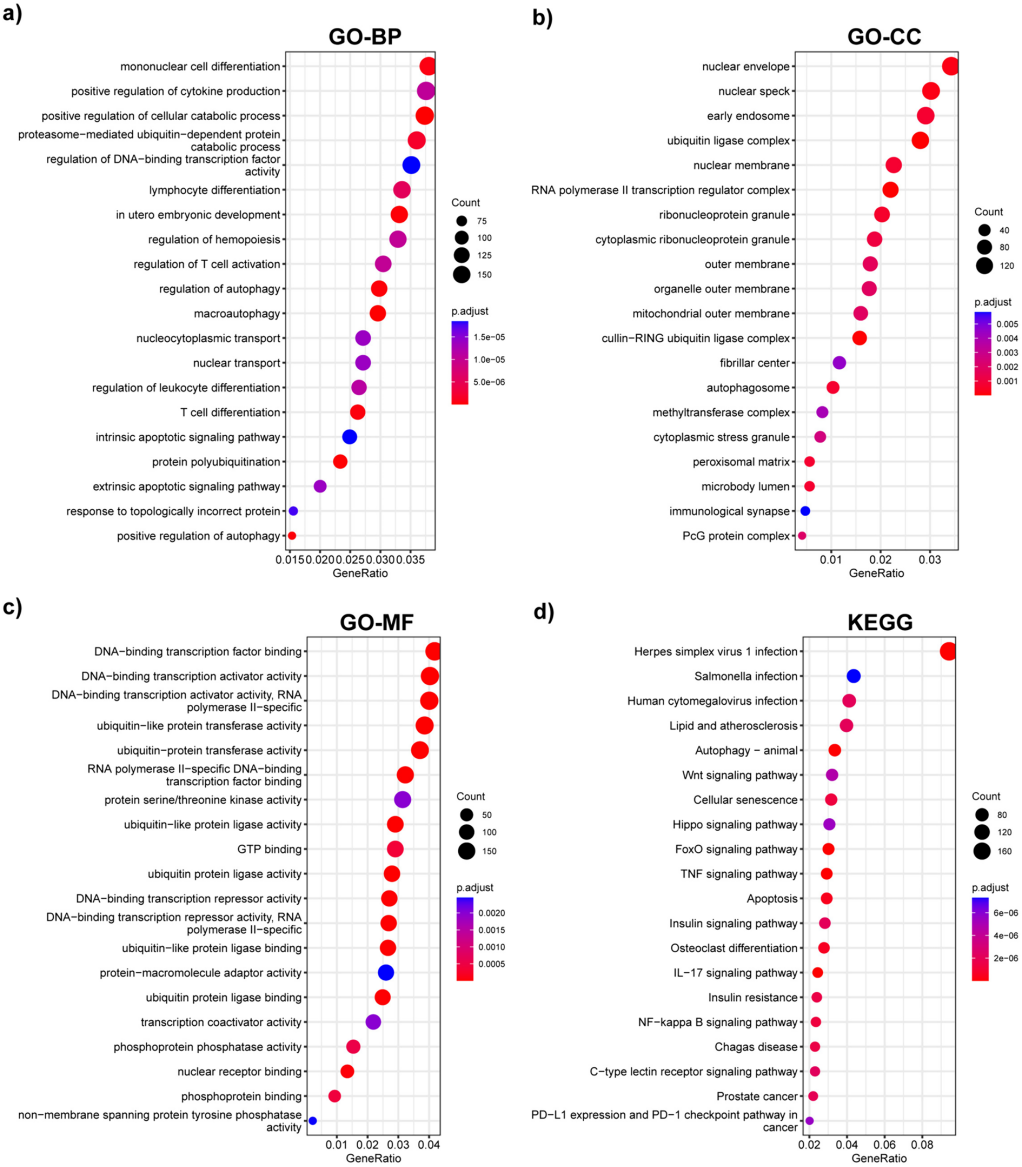
Functional analysis of DEGs. **a**–**c** GO enrichment analysis, category BP (**a**), CC (**b**), and MF (**c**) were shown in the pattern of dot plots. **d** KEGG pathway enrichment analysis. *DEGs* differentially expressed genes, *GO* gene ontology, *BP* biological process, *CC* cellular component, *MF* molecular function, *KEGG* Kyoto Encyclopedia of Genes and Genomes

To investigate the functional annotations of DEGs, we performed enrichment analyses of GO and KEGG pathways using the “clusterprofiler” package to illustrate the biological characteristics and related signaling pathways, respectively. GO terms are divided into three different categories: BP, CC, and MF (Fig. 2a–c). The top 20 significantly enriched BPs, CCs, and MFs are shown in Fig. 2a–c, respectively. In the BP category, “mononuclear cell differentiation” possessed the greatest gene ratio. In the CC category, DEGs were mostly enriched in the nuclear envelope and nuclear speck. Regarding MFs, DEGs were mainly enriched in DNA-binding transcription factor binding and DNA-binding transcription activator activity. Significantly enriched DEGs in KEGG pathways are shown in Fig. 2d. Among the top 20 pathways, the “Hippo signaling pathway,” “FoxO signaling pathway,” and “NF-kappa B signaling pathway” are known to participate in the pathophysiological process of LIRI. Moreover, multiple forms of cell death, such as “autophagy” and “apoptosis,” are involved in LIRI.

### Identification of DEMRGs

**Fig. 3:**
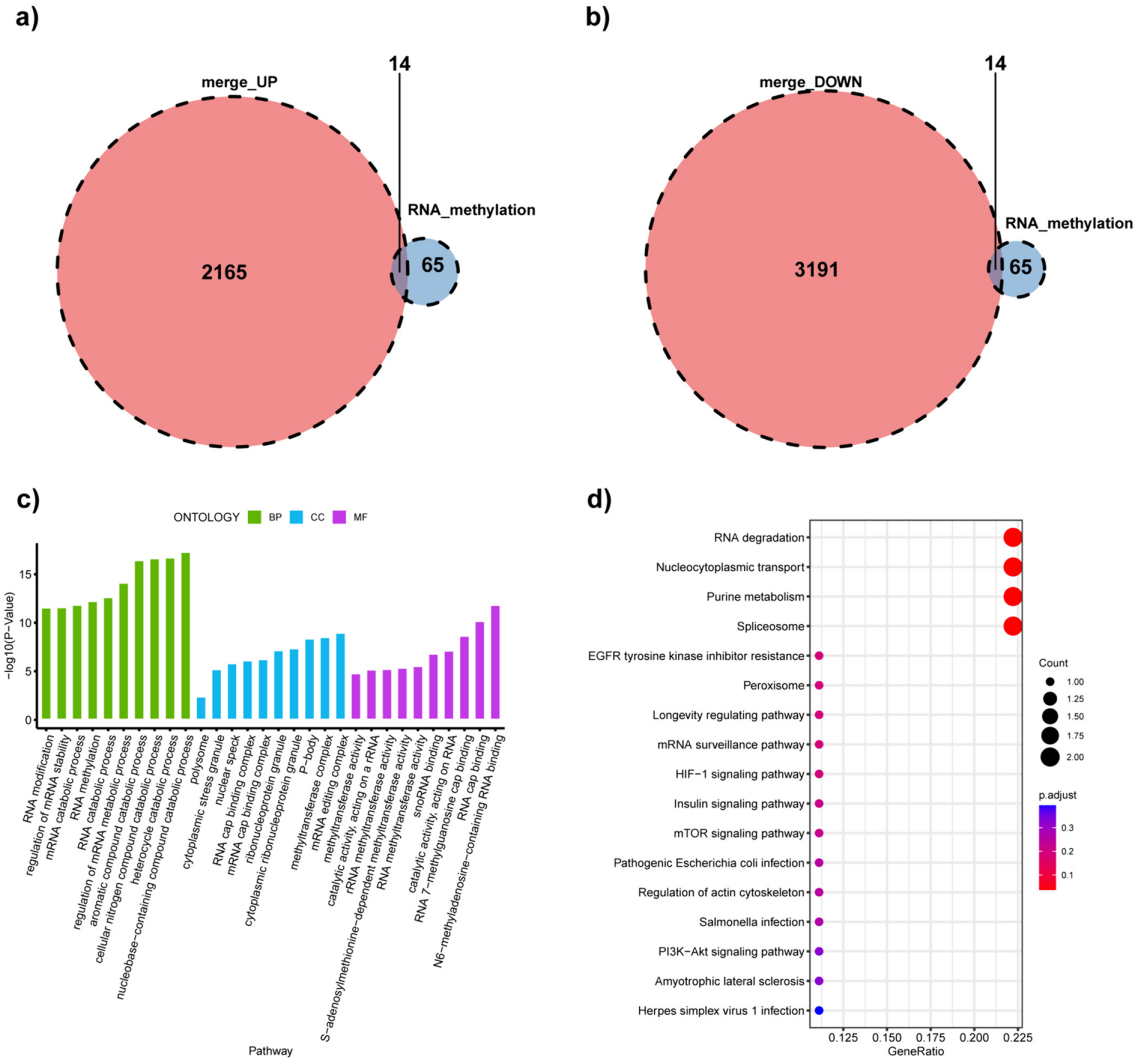
Identification of DEMRGs. **a** The Venn diagram showed 14 DEMRGs by overlapping 65 methylation-related genes and the upregulated DEGs set. **b** Another 14 DEMRGs were screened by overlapping 65 methylation-related genes and the set of down-regulated DEGs. **c** Top 10 terms significantly enriched in three GO categories by DEMRGs. **d** KEGG pathway enrichment analysis on DEMRGs. *DEMRGs* differentially expressed methylation-related genes, *DEGs* differentially expressed genes, *GO* gene ontology, *KEGG* Kyoto Encyclopedia of Genes and Genomes

To identify methylation-related genes that have a significant impact on LIRI mechanisms, we obtained 28 DEMRGs after intersecting 2165 upregulated and 3191 downregulated DEGs with 65 methylation-related genes (Fig. 3a, b). The results of functional enrichment cluster analysis of 28 DEMRGs are shown in Fig. 3c, d. In the BP category, the “nucleobase-containing compound catabolic process” constitutes the largest group. As demonstrated in KEGG pathway enrichment analysis, “RNA degradation,” “nucleocytoplasmic transport,” “purine metabolism,” and “spliceosome” predominate among these pathways. To further analyze intuitively, we constructed visualized images. A volcano plot showed the distribution of these DEMRGs (Additional file 4: Fig S1a). A heatmap of the 28 DEMRGs was constructed (Additional file 4: Fig S1b). The violin plots showed that all 28 genes were significantly differentially expressed after liver transplantation (Additional file 4: Fig S1c). The PPI network was constructed using the STRING database to interpret the interactions among DEMRGs (Additional file 5: Fig S2).

### Screening of core DEMRGs

**Fig. 4:**
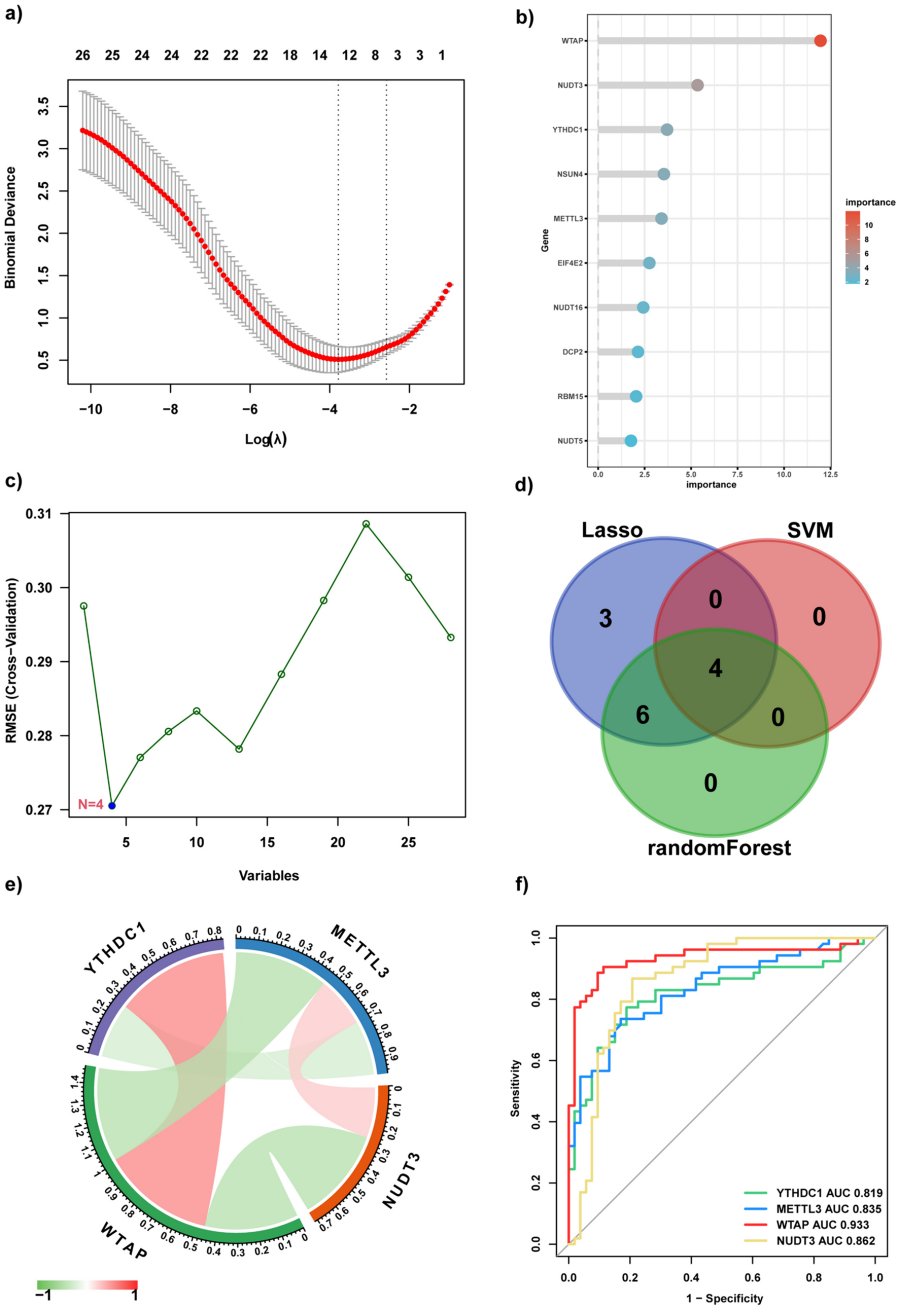
Detection of the core DEMRGs using machine learning methods. **a** The LASSO logistic regression algorithm was used to screen 13 candidate genes. **b** RF algorithm was used to pick the top 10 genes in importance order. **c** SVM-RFE was used to identify four candidate genes. **d** A Venn diagram was constructed by intersecting the candidate genes obtained by the three algorithms above. **e** The chord diagram manifested the correlation among the core DEMRGs. **f** The ROC curve for the verification of diagnostic efficacy verification. *DEMRGs* differentially expressed methylation-related genes, *LASSO* least absolute shrinkage and selection operator, *RF* random forest, *SVM-RFE* support vector machine-recursive feature elimination, *ROC* receiver operating characteristic, *AUC* area under the ROC curve

Among the 28 DEMRGs, we identified the core DEMRGs using 3 machine learning algorithms. LASSO regression algorithm was applied to identify 13 genes (Fig. 4a), RF was used to select 10 genes in descending order of importance (Fig. 4b), and SVM-RFE was employed to select 4 genes among the original 28 DEMRGs (Fig. 4c). We used a Venn diagram to determine the overlapping region of three groups of genes analyzed using the machine learning algorithms (Fig. 4d). The three algorithms identified YTH *N*6-methyladenosine RNA binding protein C1 (*YTHDC1*), *METTL3*, Wilms tumor 1-associated protein (WTAP), and Nudix hydrolase 3 (*NUDT3*) as overlapping DEMRGs at the core. Correlation analysis of the four genes is displayed in the chord diagram, which was constructed using the “circlize” package. In this diagram, the red lines represented a positive correlation, and the genes connected by green lines represented a negative correlation. Moreover, the darker the color, the stronger the correlation (Fig. 4e). To assess the predictive ability of LIRI, we constructed the gene prediction model using the “pROC” package (Fig. 4f). A higher AUC value indicates superior diagnostic performance. The ROC curves for *YTHDC1*, *METTL3*, *WTAP*, and *NUDT3* demonstrated a reasonably satisfactory prediction accuracy, with AUCs of 0.819, 0.835, 0.933, and 0.862, respectively. As shown in Additional file 6: Fig S3, following LIRI, a notable increase was observed in the expression of *YTHDC1* and *WTAP*, whereas the expression of *METTL3* and *NUDT3* decreased significantly.

### Validation of core DEMRG expression and cytological experiments

**Fig. 5:**
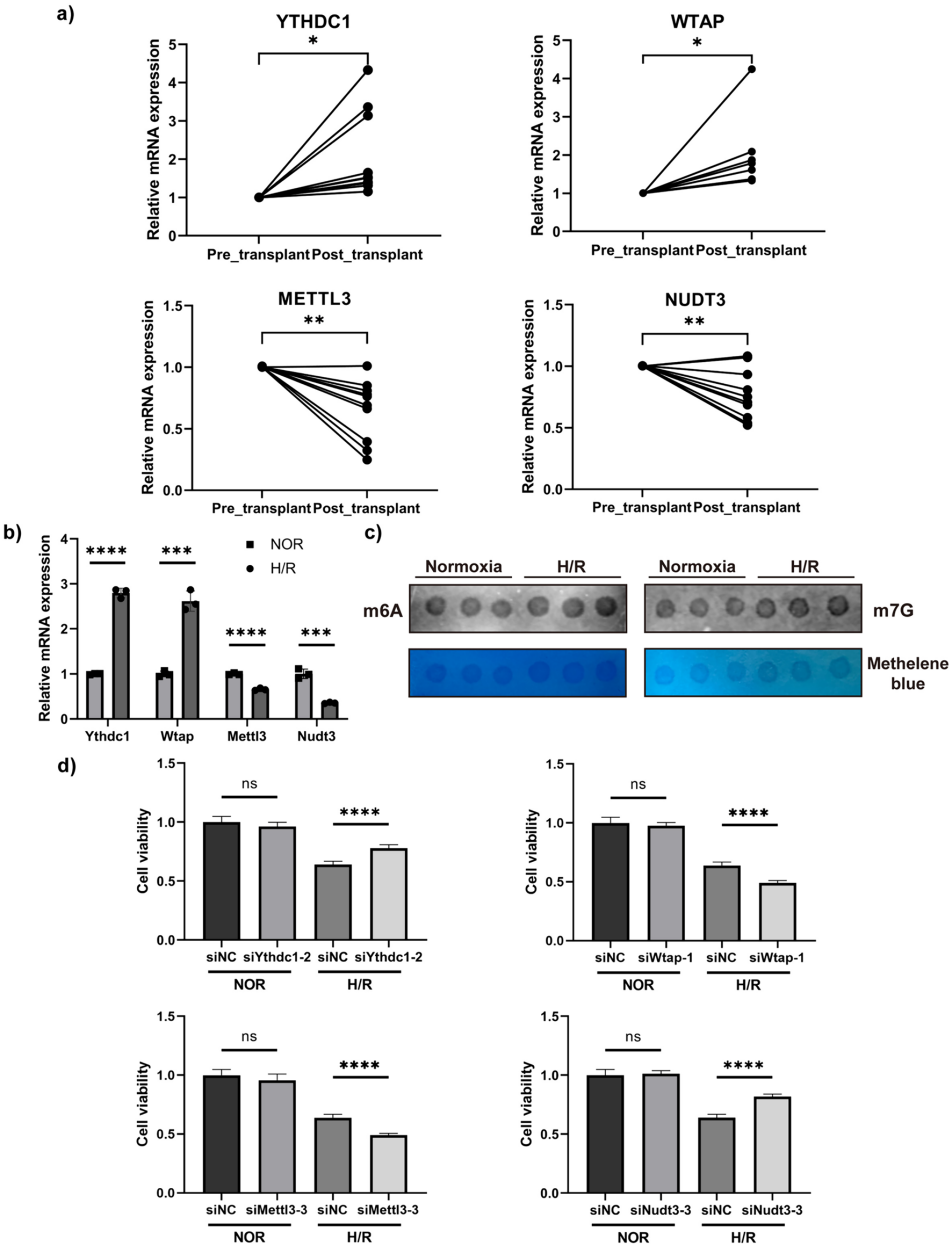
Expression and function validation of core DEMRGs. **a** The mRNA expression level of core DEMRGs in clinical liver transplantation samples. **b** The mRNA expression level of core DEMRGs in hepatocytes after treated by H/R. **c** m6A and m7G level detection by using Dot Blot in vitro. **d** The viability of cells in each group was evaluated by the CCK-8 assay. The* P*-values were shown as: *P < 0.05, **P < 0.01, ***P < 0.001, ****P < 0.0001. *ns* non-significant, *DEMRGs* differentially expressed methylation-related genes, *H/R* hypoxia/reoxygenation

We examined the changes in the expression of core DEMRGs in 10 pairs of liver transplantation samples. The results were consistent with those of transcriptome analysis. Compared with liver tissue before transplantation, a significant increase was observed in the mRNA expression of *YTHDC1* and *WTAP* post-transplantation, whereas the mRNA expression of *METTL3* and *NUDT3* decreased significantly (Fig. 5a). Subsequently, we isolated and cultured primary mouse hepatocytes and established a H/R model, with cells undergoing 6 h of hypoxia followed by 6 h of reoxygenation. After extracting RNA from cells, we compared the expression of the core DEMRGs before and after H/R stimulation, and the results indicated that the changes in the expression of the four DEMRGs were consistent with those observed in the clinical samples (Fig. 5b). Among the four types of methylation modifications identified in our previous analysis, only genes related to m6A or m7G showed significant changes. Therefore, we determined whether there were alterations at the level of m6A and m7G methylation in hepatocytes after LIRI. We performed dot blot assays to detect changes in m6A and m7G methylation in hepatocytes. The results revealed a significant increase in the overall levels of m6A and m7G methylation following H/R stimulation (Fig. 5c). Next, we performed functional validation of each core DEMRG. Three siRNAs were designed for each gene, and RT-qPCR was used to determine the siRNA that inhibited gene expression most effectively (Additional file 7: Fig S4). Next, we evaluated the effect of each core DEMRG on cell viability using the CCK-8 assay. We demonstrated that altering the expression of core DEMRGs within cells under normoxic conditions did not significantly affect cell viability. Subsequently, we subjected the cells to H/R stimulation and then performed CCK-8 assay. The results indicated that after reducing the expression of *YTHDC1* or *NUDT3*, cell viability increased significantly in the siRNA group compared with that in the NC group. Conversely, decreasing the expression of *METTL3* or *WTAP* resulted in significantly lower cell viability in the siRNA group than in the NC group (Fig. 5d).

### Assessment of infiltration of immune cells

**Fig. 6:**
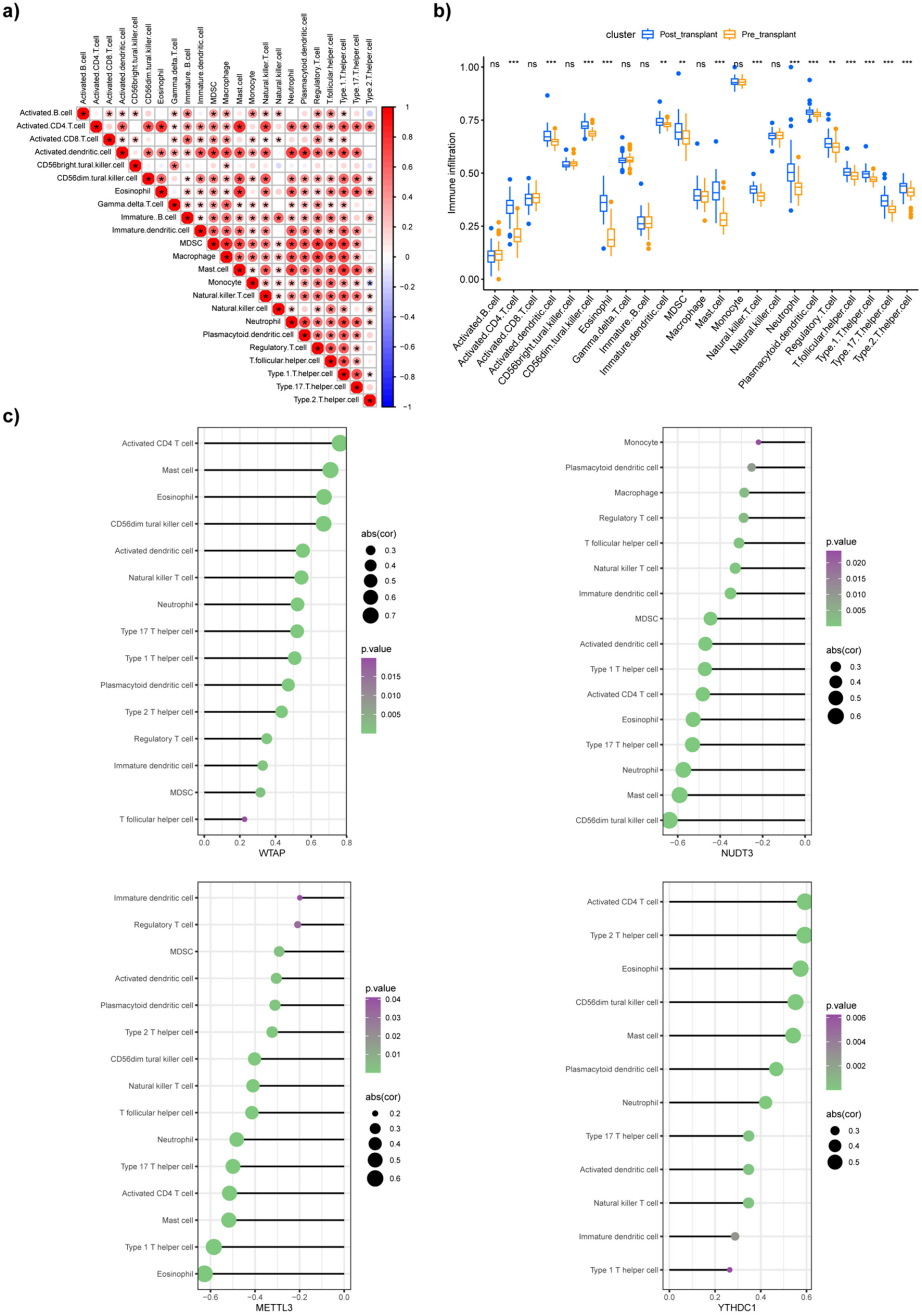
The normalized data set for the evaluation of the degree of immune cell infiltration. **a** Correlation coefficient diagram showing the relationship among immune cells. The **b** box plot showed the differences in immune cell infiltration between the pre-transplant and post-transplant groups. **c** Lollipop diagrams showed the correlation between the core DEMRGs and immune cells. *P < 0.05, only immune cells with* P* < 0.05 are shown. *DEMRGs* differentially expressed methylation-related genes

The ssGSEA method from the “GSVA” R package was used to assess the correlation among immune cells, with a darker red color representing a greater association between any two types of cells (Fig. 6a). We compared immune cell infiltration between pre- and post-transplant samples according to ssGSEA scores. As shown in the box plot, activated CD4 T cells, activated dendritic cells, mast cells, CD65dim natural killer cells, natural killer T cells, regulatory T cells, T helper 1 cells, T helper 2 cells, T helper 17 cells, eosinophils, and neutrophils were more abundant in the post-transplant group than in the pre-transplant group (Fig. 6b). We then investigated the potential association between the expression levels of each of the core DEMRGs and 23 types of immune cells (Fig. 6c). The lollipop diagrams indicated that the expression of *WTAP* and *YTHDC1* was significantly positively correlated with the infiltration levels of activated CD4 T cells, mast cells, eosinophils, and CD65dim natural killer cells. In contrast, the expression of *NUDT3* was negatively correlated with the infiltration levels of CD65dim natural killer cells, mast cells, eosinophils, and neutrophils. Additionally, the expression of *METTL3* was negatively correlated with the infiltration levels of activated CD4 T cells, eosinophils, T helper 1 cells, mast cells, and activated dendritic cells.

### Further research on core DEMRGs

**Fig. 7:**
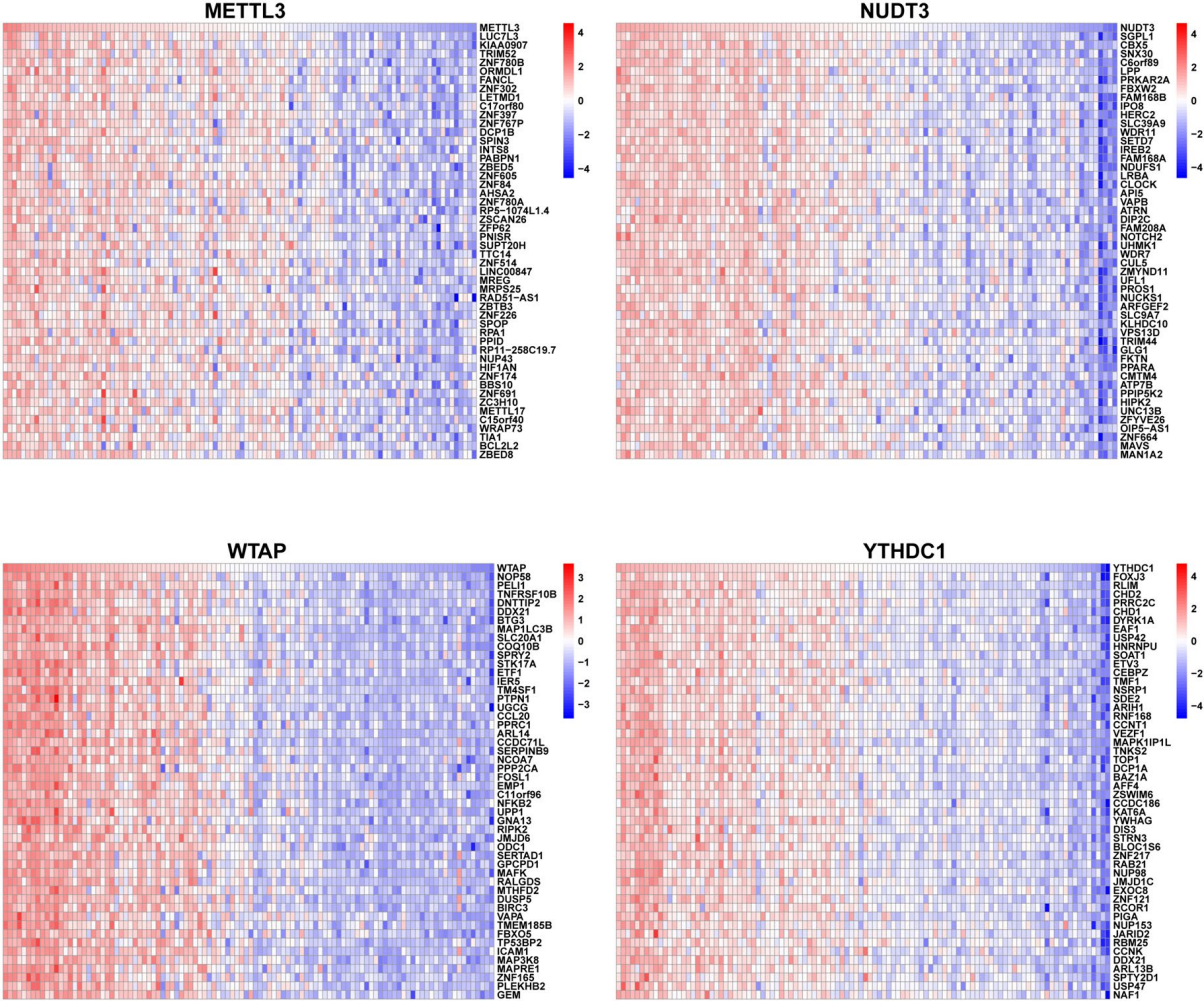
Heat map of the top 50 positively correlated genes in the core DEMRGs. *DEMRGs* differentially expressed methylation-related genes

**Fig. 8:**
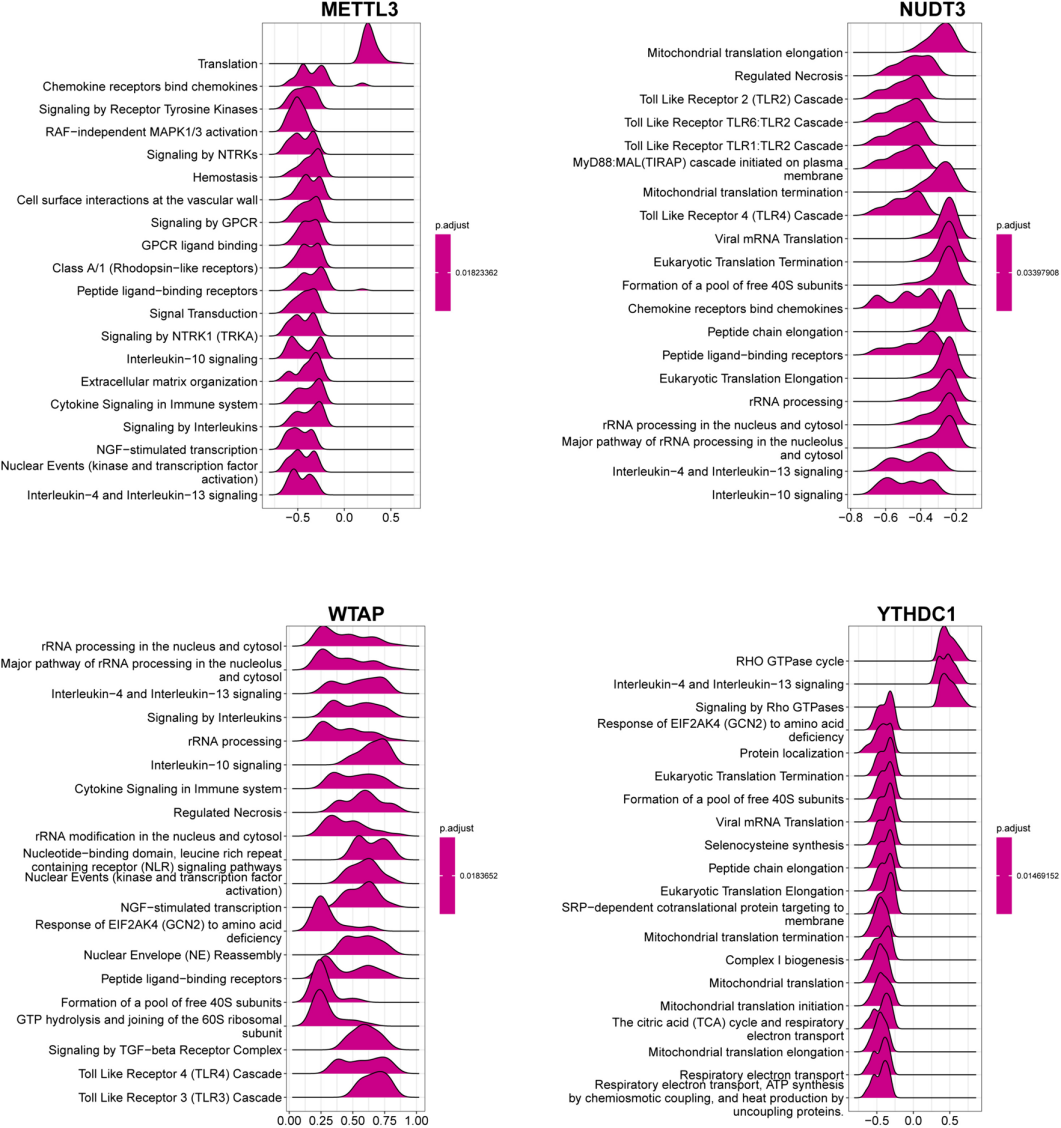
Gene set enrichment analysis of single-core DEMRGs based on the REACTOME database. *DEMRGs* differentially expressed methylation-related genes

To explore the possible function of core DEMRGs in LIRI, including the interrelated pathways, we performed a correlation analysis between each of the core DEMRGs and the rest of the genes. Then, we revealed the top 50 genes most positively associated with the core DEMRGs by constructing heatmaps (Fig. 7). GSEA based on REACTOME was performed to determine the significant pathways mainly associated with the mechanisms of LIRI. Ridgeline plots of the top 20 pathways obtained using the “clusterProfiler” R package are displayed in Fig. 8. With an adjusted *P*-value of 0.01823362, the enrichment of Interleukin-10 signaling, Interleukin-4, and Interleukin-13 signaling, Signaling by interleukins, and Cytokine signaling in the immune system was negatively associated with the expression of *METTL3*. Similarly, the enrichment of Toll-like receptor 2 cascade, Toll-like receptor 4 cascade, interleukin-10 signaling, interleukin-4, and interleukin-13 signaling was significantly negatively associated with the expression of *NUDT3*. In contrast, the enrichment of interleukin-10 signaling, interleukin-4, interleukin-13 signaling, cytokine signaling in the immune system, Toll-like receptor 4 cascade, and Toll-like receptor 3 cascade was significantly associated with the expression of *WTAP*. Furthermore, the enrichment of mitochondrial translation initiation, mitochondrial translation, mitochondrial translation termination, and respiratory electron elongation was significantly negatively associated with the expression of *YTHDC1*. These results suggest that the core DEMRGs are associated with many pathways in the progression of LIRI, especially immune-related and oxidative metabolism pathways. We then predicted the upstream miRNAs and transcription factors of the four core DEMRGs using the RegNetwork data repository and constructed the network using Cytoscape (Additional file 8: Fig S5). As shown in the network, activating the transcription factor family might be the crucial upstream transcription factor of *YTHDC1* and *NUDT3*. Additionally, the E2F family showed potential as the upstream transcription factors of *YTHDC1* and *METTL3*. Seven miRNAs, including hsa-miR-181a, hsa-miR-181b, hsa-miR-181c, hsa-miR-543, hsa-miR-141, hsa-miR-200a, and hsa-miR-451, showed the possibility of being present upstream of *WTAP* and *YTHDC1*. Among all miRNAs and transcription factors, MYC showed the best match as the predictive factor, which could be an upstream transcription factor of *YTHDC1*, *NUDT3*, and *METTL3*.

## Discussion

In general, LIRI is caused by multiple liver surgeries or various forms of trauma. It inflicts liver function through intricate biological processes, including but not limited to oxidative stress, calcium overload, apoptosis, pyroptosis, and ferroptosis. However, despite complicated and specific mechanisms, LIRI always starts with activated Kupffer cells, followed by the process of immune infiltration [3, 11]. Studies have reported that RNA modification, particularly represented by m6A, m5C, m1A, and m7G, plays a significant role in various biological progresses [40]. Immune responses, including innate and adaptive immune responses, are regulated via RNA modification by altering the modified bases [41, 42]. Although the partial correlation between RNA modification and LIRI has been shown, it remains unclear whether it is affected by immune processes and specific targets [43]. To further illustrate how RNA methylation modifications participate in the immune modulatory mechanisms of LIRI, we performed a series of analyses systematically.

In this study, according to the etiology of liver ischemia and reperfusion, we found two applicable sets of liver transplantation samples from the GEO database and then merged them. We removed the batch effect for the following analyses. We identified DEGs with an adjusted *P*-value of < 0.05 and subsequently performed GO and KEGG enrichment analyses. The BP category in GO analysis showed that all DEGs were mainly associated with multiple immune cell differentiation, regulation of cytokine production, regulation of T cell activation, and regulation of autophagy, indicating immunological processes. In the CC category, most terms were related to the nuclear envelope, ribonucleoprotein granule, and organelle outer membrane, including the mitochondrial outer membrane, suggesting that DEGs participated in the transcription and translation processes within cells. In addition, the MF category demonstrated that the DEG functions were largely concentrated in DNA-binding transcription activator activity, ubiquitin-like protein transferase activity, and ubiquitin-like protein ligase activity, suggesting that the cell death forms derived from ubiquitination were mainly involved in LIRI. KEGG enrichment analysis revealed that DEGs possessed some correlation with Herpes simplex virus 1 infection, autophagy, apoptosis, and several canonical signaling pathways, including the Wnt signaling pathway, FoxO signaling pathway, IL-17 signaling pathway, and NF-kappa B signaling pathway. We obtained 28 DEMRGs after overlapping the DEG sets and 65 methylation-related genes and then constructed a PPI network of the core genes using the STRING database. Three types of machine learning techniques were applied to identify the core DEMRGs, each of which had a unique method for screening potential makers. Notably, *YTHDC1*, *METTL3*, *WTAP*, and *NUDT3* were selected, and as all four AUC values were > 0.8, the predictive model showed satisfactory predictive performance. The ssGSEA method was used to analyze the immune infiltration process that occurs during LIRI.

As the pivotal core of the inflammatory cytotoxic cycle in LIRI, the immune infiltration process is undoubtedly characterized by sensitive and explorable changes. Neutrophils, being the primary line of defense against invading pathogens, are mobilized to the site of ischemia/reperfusion and considered a biomarker for LIRI [44]. CD4 T cells are considered essential inflammatory mediators in the LIRI process [45]. Activated CD4 T cells were shown to activate innate immunity and amplify the subsequent cascade reaction [46]. Furthermore, original CD4 T cells undergo differentiation into diverse T cell subtypes, such as regulatory T, T helper 1, T helper 2, and T helper 17 cells. These subtypes play different roles in modifying inflammatory responses [47]. Another type of CD4 T cells—natural killer T cells—recruit and activate natural killer cells directly and play a role in the response of pro- or anti-inflammation in LIRI according to different subsets [48, 49]. Recently, eosinophils have been reported to accumulate in the liver after LIRI and possess a fairly strong protective function [50].

METTL3, METTL14, and WTAP together form the m6A methyltransferase complex that regulates the m6A methylation level of RNA in cells [51]. The results indicated significant alterations in the expression levels of *METTL3* and *WTAP*, which exhibited certain protective effects on hepatocytes during LIRI. In addition, a significant increase in total m6A levels was observed in hepatocytes following H/R stimulation, which suggests that METTL3 and WTAP may be the primary reason for the increased m6A levels during LIRI, which potentially influences the pathophysiology of LIRI. Research has shown that METTL3 expression level alters the proliferation and differentiation of T cells via the IL-7 signaling pathway, which is consistent with our results [52]. Furthermore, by altering the mRNA m6A methylation levels, METTL3 promotes the function of DC cells, which play a different role in LIRI, depending on the location of DC cells in vivo [53, 54]. METTL3-mediated m6A modification primarily affects IL-13-encoding stability of the mRNA in mast cells [55]. Additionally, evidence suggests that degranulation of gastrointestinal mast cells in rats is positively correlated with LIRI-induced damage [56]. Regarding WTAP, Wang et al. [57] revealed a strong link between WTAP and myocardial IRI. They demonstrated that knocking down *WTAP* protects cardiomyocytes from endoplasmic reticulum stress by decreasing the stability of activating transcription factor 4 (ATF4) mRNA. However, the mechanism underlying the role of WTAP in LIRI has not yet been reported. In particular, increased WTAP expression enhances YTH domain-containing family protein 1-mediated translation efficiency of forkhead box O1, which results in upregulated protein levels, ultimately promoting regulatory T cell differentiation and function [58]. Furthermore, WTAP-induced m6A methylation is crucial for the activation of TCR-dependent CD4^+^ and CD8^+^ T cells as well as the survival of activated T cells [59]. YTHDC1, one of the classic readers of m6A, together with WTAP has been demonstrated to regulate the immune microenvironment in ischemic cardiomyopathy [60]. Furthermore, YTHDC1 alleviates ischemic stroke by promoting the degradation of phosphatase and tensin homolog mRNA following Akt phosphorylation [61]. To date, evidence regarding the influence of YTHDC1 on immune infiltration is limited, with only a few studies suggesting its ability to modulate immune response in cancer [62]. Our results provide further evidence regarding the association between YTHDC1 and immune infiltration, which is mediated by activated CD4 T cells and neutrophils during LIRI.

In addition to m6A, we identified *NUDT3*, one of the related genes of m7G, as a core DEMRG, but no other characteristic genes were detected in the group of genes related to m1A or m5C. m7G was first discovered at the 5′-cap of mRNAs; it plays an essential role in stabilizing transcripts against exonucleolytic degradation [63]. Cap modification also affects several stages of mRNA lifespan, including transcription elongation [64], pre-mRNA splicing [65], and translation [66]. m7G sites were also detected in transfer RNA [67] and ribosomal RNA [68] and were discovered internally within mRNA in 2019 [69]. Unlike the well-studied pattern of the modification, i.e., regulating the level of methylation with “writers” and “erasers” and executing with “readers,” methyltransferase-like 1 (METTL1) and WD repeat domain 4 (WDR4), together forming a functional methyltransferase complex, have been perceived to be the only two clear factors serving as the “writers” of m7G modification. However, in July 2023, Quaking proteins have been reported as the first “reader” of m7G modification [70, 71]. No prior studies have examined the potential relationship between m7G modification and LIRI. The current study revealed a significant elevation in total m7G levels in hepatocytes following H/R stimulation. This suggests dynamic alterations in m7G methylation and demethylation during LIRI, which warrants further investigation. Similarly, postischemic angiogenesis could be enhanced via METTL1 by promoting the translation of vascular endothelial growth factor A mRNA [24]. Wang et al*.* showed that *METTL1* ablation in fibroblasts decreases the expression of m7G methylated fibrotic genes, thereby alleviating myocardial infarction-induced cardiac fibrosis [25]. The Nudix superfamily, a series of hydrolases that possess a conserved nucleoside diphosphate linked to another moiety X (Nudix), serving as divalent cation-regulated enzymes that hydrolyze various dinucleotides and inositol pyrophosphates (PP-InsPs), contains 8 of 22 Nudix proteins in the human genome, namely, Nudt2, Nudt3, Nudt12, Nudt15, Nudt16, Nudt17, Nudt19, and Dcp2 (Nudt20), and possesses the ability to decap RNA [72, 73]. Notably, Nudt3 was shown to play a crucial role in maintaining cell viability during oxidative stress, acting as a Zn^2+^-dependent polyphosphate hydrolase both in vitro and in vivo [74]. Furthermore, the study revealed the potential protective effect of downregulating NUDT3 in LIRI and its robust association with natural killer cells, neutrophils, and T helper 17 cells. m7G modification associated with NUDT3 may serve as a promising and innovative therapeutic target after additional validation.

This study has some limitations, despite the comprehensive analysis of methylation modification in LIRI and the relevant immune infiltration. First, it was a retrospective study based on public databases. A validation study with a larger clinical sample size is warranted to further demonstrate the potential of core DEMRGs in predicting LIRI progression. Second, it is necessary to further investigate whether the crucial genes related to m1A or m5C play roles in LIRI, as the current study only identified the central genes related to m6A and m7G.

## Conclusions

This study revealed that the methylation-related genes *YTHDC1*, *METTL3*, *WTAP*, and *NUDT3* emerge as core DEMRGs, indicating their distinct biological pivotal roles as diagnostic markers for LIRI. Furthermore, the analyses underscored the potential involvement of immune cells in the progression of LIRI, with *YTHDC1*, *METTL3*, *WTAP*, and *NUDT3* showing numerous associations across a diverse array of immune cell types. These findings highlighted that immune cells tend to exert a significant influence on the advancement of LIRI. Thus, a comprehensive exploration of these immune cells could offer valuable insights into determining targets for immunotherapy and optimizing immunomodulatory strategies to improve post-liver transplantation recovery.

### Supplementary Information

Additional file 1: Table S1. The details of the GEO datasets used to analysis.

Additional file 2: Table S2. siRNA sequences.

Additional file 3: Table S3. The primer sequences for RT-qPCR.

Additional file 4: Fig. S1. DEMRGs between the pre-transplant and post-transplant groups. a, b DEMRGs were shown in the volcano plot (a) and Heatmap (b). c Differential expression of DEMRGs in GEO gene sets. DEMRGs: differentially expressed methylation-related genes*.* The* P*-values were shown as: *P < 0.05, **P < 0.01, ***P < 0.001, ****P < 0.0001.

Additional file 5: Fig. S2. The protein–protein interaction network of DEMRGs. DEMRGs: differentially expressed methylation-related genes.

Additional file 6: Fig. S3. The expression level of core DEMRGs in datasets. The* P*-values were shown as: ****P < 0.0001.

Additional file 7: Fig. S4. Efficiency verification of RT-qPCR for siRNAs targeting core DEMRGs. The* P*-values were shown as: *P < 0.05, **P < 0.01, ****P < 0.0001.

Additional file 8: Fig. S5. Construction of a network consisting of the potential upstream miRNAs and transcription factors of core DEMRGs.

## Data Availability

The original contributions presented in the study are included in the article/Supplementary Information, further inquiries can be directed to the corresponding authors.

## Abbreviations

ATF4: Activating transcription factor 4
AUC: Area under the ROC curve
BP: Biological process
cDNA: Complementary DNA
CC: Cellular component
DEMRGs: Differentially expressed methylation-related genes
DEGs: Differentially expressed genes
FTO: Fat mass and obesity-associated protein
FOXO1: Forkhead box O1
GEO: Gene Expression Omnibus
GO: Gene ontology
GSEA: Gene set enrichment analysis
HBSS: Hanks Balanced Salt Solutions
H/R: Hypoxia/reoxygenation
IRI: Ischemia–reperfusion injury
KEGG: Kyoto Encyclopedia of Genes and Genomes
LASSO: Least absolute shrinkage and selection operator
LIRI: Liver IRI
m1A: *N*1-Methyladenosine
m5C: 5-Methylcytosine
m6A: *N*6-Methyladenosine
m7G: 7-Methylguanine
METTL1: Methyltransferase-like 1
METTL3: Methyltransferase-like 3
MF: Molecular function
MTC: Methyltransferase complex
NC: Negative control
NUDT3: Nudix hydrolase 3
Nudix: Nucleoside diphosphate linked to another moiety X
PPI: Protein–protein interaction
PTEN: Phosphatase and tensin homolog
QKIs: Quaking proteins
RegNetwork: Regulatory Network Repository of Transcription Factor and microRNA-Mediated Gene Regulations
RF: Random forests
ROC: Receiver operating characteristic
RT-qPCR: Real-Time Quantitative Polymerase Chain Reaction
siRNA: Small interfering RNA
ssGSEA: Single-sample gene set enrichment analysis
SVM-RFE: Support vector machine-recursive feature elimination
STRING: Search Tool for the Retrieval of Interacting Genes
VEGFA: Vascular endothelial growth factor A
WDR4: WD repeat domain 4
WTAP: Wilms tumor 1-associated protein
YTHDC1: YTH *N*6-methyladenosine RNA-binding protein C1
YTHDF1: YTH domain-containing family protein 1
