# Supplementary material for: A comprehensive analysis of m6A/m7G/m5C/m1A-related gene expression and immune infiltration in liver ischemia–reperfusion injury by integrating bioinformatics and machine learning algorithms
Source: Eur J Med Res. 2024 Jun 13;29:326. doi: 10.1186/s40001-024-01928-y (PMC11170855; doi:10.1186/s40001-024-01928-y)
Supplement: Supplementary file 1 — Additional file 1: Table S1. The details of the GEO datasets used to analysis. [file 40001_2024_1928_MOESM1_ESM.docx]

**Additional file 1: Table S1.** **The details of the GEO datasets used to analysis**

| GEO Dataset | Platform | Type of tissue | Pre-transplant | Post-transplant |
| --- | --- | --- | --- | --- |
| GSE12720 | Affymetrix Human Genome U133 Plus 2.0 Array | Liver biopsy tissue | 13 | 13 |
| GSE151648 | Illumina HiSeq 3000 | Liver biopsy tissue | 40 | 40 |
