# Supplementary material for: A comprehensive analysis of m6A/m7G/m5C/m1A-related gene expression and immune infiltration in liver ischemia–reperfusion injury by integrating bioinformatics and machine learning algorithms
Source: Eur J Med Res. 2024 Jun 13;29:326. doi: 10.1186/s40001-024-01928-y (PMC11170855; doi:10.1186/s40001-024-01928-y)
Supplement: Supplementary file 2 — Additional file 2: Table S2. siRNA sequences. [file 40001_2024_1928_MOESM2_ESM.docx]

**Additional file 2: Table S2. siRNA sequences**

| Number | siRNA name | Gene ID | Species | Sequence (5′–3′) |
| --- | --- | --- | --- | --- |
| 1 | siMettl3-1 | 56335 | Mus musculus | GCACAUCCUACUCUUGUAATT |
| 2 | siMettl3-2 | 56335 | Mus musculus | CGAUGUUGAUCUGGAGAUATT |
| 3 | siMettl3-3 | 56335 | Mus musculus | GGUUCGUUCCACCAGUCAUTT |
| 4 | siNudt3-1 | 56409 | Mus musculus | CCAUCCAGACCGAUGGAUUTT |
| 5 | siNudt3-2 | 56409 | Mus musculus | GCACAGGACCUACGUGUAUTT |
| 6 | siNudt3-3 | 56409 | Mus musculus | GGAAGAGAGAGUGGUUUAATT |
| 7 | siYthdc1-1 | 231386 | Mus musculus | GGAGGAAGAUGUAGAGGAATT |
| 8 | siYthdc1-2 | 231386 | Mus musculus | GGAUGAAGAGGAAGAAGAATT |
| 9 | siYthdc1-3 | 231386 | Mus musculus | CGAGAAAGAGAGAGAGAAATT |
| 10 | siWtap-1 | 60532 | Mus musculus | ATGGCAAGAGATGAGTTAATT |
| 11 | siWtap-2 | 60532 | Mus musculus | GGCAAGTACACAGATCTTAAC |
| 12 | siWtap-3 | 60532 | Mus musculus | GCAAGAGTGTACTACTCAAAT |
| 13 | siNC |  | Mus musculus | UUCUCCGAACGUGUCACGUTT |
