# Supplementary material for: A comprehensive analysis of m6A/m7G/m5C/m1A-related gene expression and immune infiltration in liver ischemia–reperfusion injury by integrating bioinformatics and machine learning algorithms
Source: Eur J Med Res. 2024 Jun 13;29:326. doi: 10.1186/s40001-024-01928-y (PMC11170855; doi:10.1186/s40001-024-01928-y)
Supplement: Supplementary file 3 — Additional file 3: Table S3. The primer sequences for RT-qPCR. [file 40001_2024_1928_MOESM3_ESM.docx]

**Additional file 3: Table S3. The primer sequences for RT-qPCR.**

| Number | Primer name | Gene ID | Species | Sequence (5′–3′) |
| --- | --- | --- | --- | --- |
| 1 | YTHDC1-F | 91746 | Homo sapiens | AACTGGTTTCTAAGCCACTGAGC |
| 2 | YTHDC1-R | 91746 | Homo sapiens | GGAGGCACTACTTGATAGACGA |
| 3 | NUDT3-F | 11165 | Homo sapiens | GAAGCACAGGACGTATGTCTATG |
| 4 | NUDT3-R | 11165 | Homo sapiens | CTGCACGGGTTTGTGATACTG |
| 5 | WTAP-F | 9589 | Homo sapiens | CTTCCCAAGAAGGTTCGATTGA |
| 6 | WTAP-R | 9589 | Homo sapiens | TCAGACTCTCTTAGGCCAGTTAC |
| 7 | METTL3-F | 56339 | Homo sapiens | TTGTCTCCAACCTTCCGTAGT |
| 8 | METTL3-R | 56339 | Homo sapiens | CCAGATCAGAGAGGTGGTGTAG |
| 9 | GAPDH-F | 2597 | Homo sapiens | GGAGCGAGATCCCTCCAAAAT |
| 10 | GAPDH-R | 2597 | Homo sapiens | GGCTGTTGTCATACTTCTCATGG |
| 11 | Ythdc1-F | 231386 | Mus musculus | GTCCACATTGCCTGTAAATGAGA |
| 12 | Ythdc1-R | 231386 | Mus musculus | GGAAGCACCCAGTGTATAGGA |
| 13 | Nudt3-F | 56409 | Mus musculus | CTGTCCGGGAAGTGTGTGAG |
| 14 | Nudt3-R | 56409 | Mus musculus | CATACACGTAGGTCCTGTGCT |
| 15 | Wtap-F | 60532 | Mus musculus | GAACCTCTTCCTAAAAAGGTCCG |
| 16 | Wtap-R | 60532 | Mus musculus | TTAACTCATCCCGTGCCATAAC |
| 17 | Mettl3-F | 56335 | Mus musculus | CTGGGCACTTGGATTTAAGGAA |
| 18 | Mettl3-R | 56335 | Mus musculus | TGAGAGGTGGTGTAGCAACTT |
| 19 | Gapdh-F | 14433 | Mus musculus | AGGTCGGTGTGAACGGATTTG |
| 20 | Gapdh-R | 14433 | Mus musculus | TGTAGACCATGTAGTTGAGGTCA |
