# Supplementary figures and images for: A comprehensive analysis of m6A/m7G/m5C/m1A-related gene expression and immune infiltration in liver ischemia–reperfusion injury by integrating bioinformatics and machine learning algorithms
Source: Eur J Med Res. 2024 Jun 13;29:326. doi: 10.1186/s40001-024-01928-y (PMC11170855; doi:10.1186/s40001-024-01928-y)

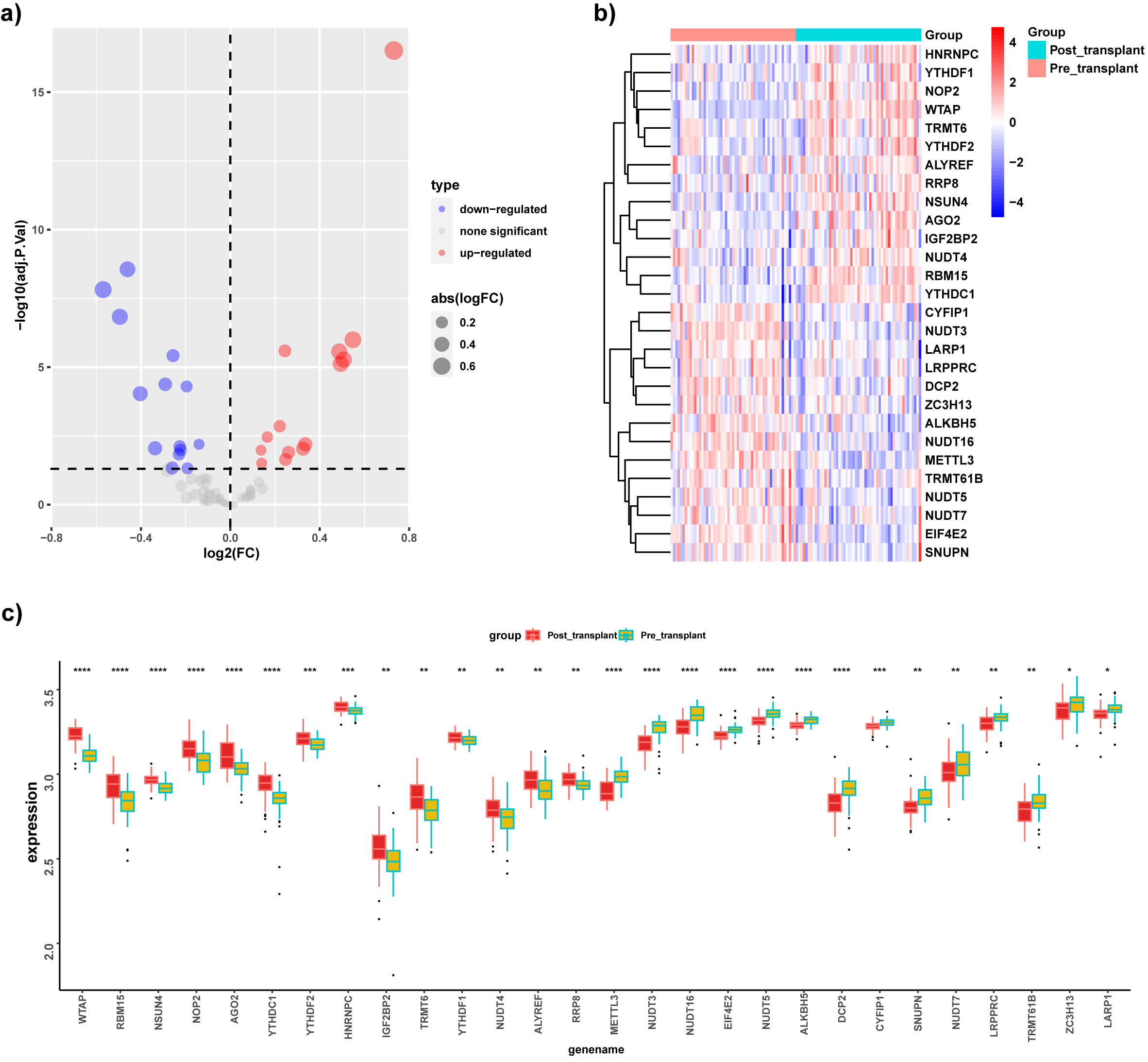

Supplement: Supplementary file 4 — Additional file 4: Fig. S1. DEMRGs between the pre-transplant and post-transplant groups. a, b DEMRGs were shown in the volcano plot (a) and Heatmap (b). c Differential expression of DEMRGs in GEO gene sets. DEMRGs: differentially expressed methylation-related genes. The P-values were shown as: *P < 0.05, **P < 0.01, ***P < 0.001, ****P < 0.0001. [file 40001_2024_1928_MOESM4_ESM.tif]

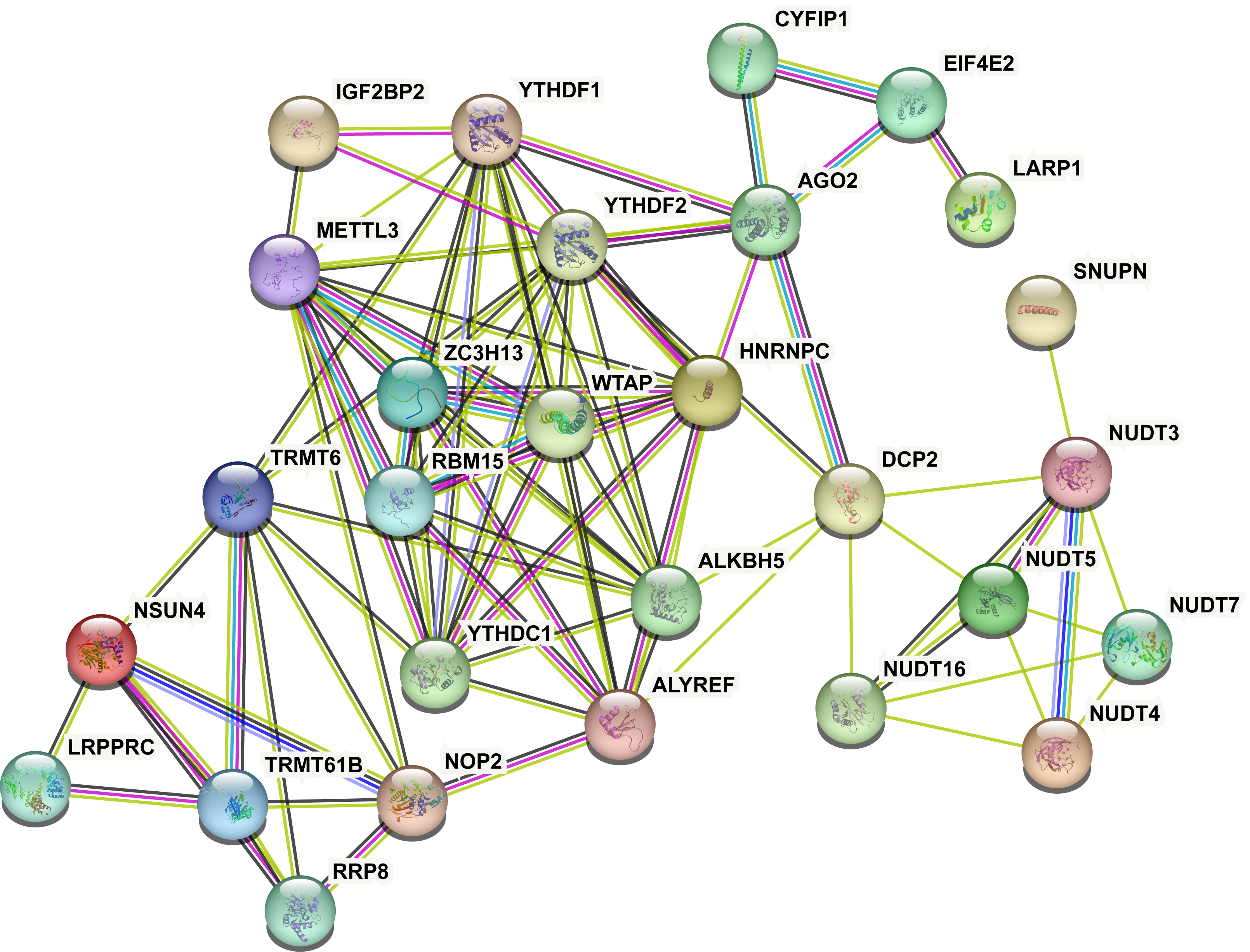

Supplement: Supplementary file 5 — Additional file 5: Fig. S2. The protein–protein interaction network of DEMRGs. DEMRGs: differentially expressed methylation-related genes. [file 40001_2024_1928_MOESM5_ESM.tif]

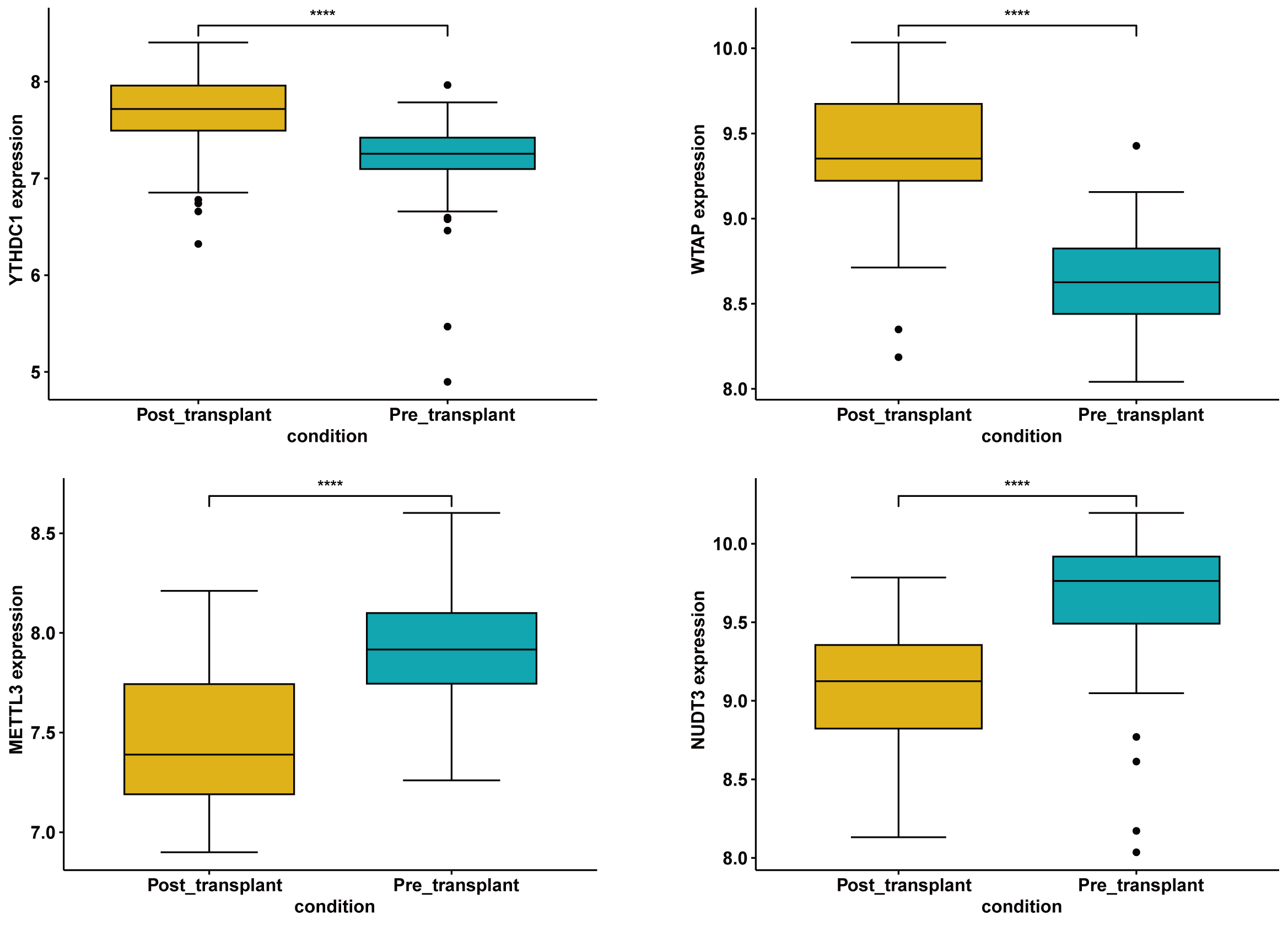

Supplement: Supplementary file 6 — Additional file 6: Fig. S3. The expression level of core DEMRGs in datasets. The P-values were shown as: ****P < 0.0001. [file 40001_2024_1928_MOESM6_ESM.tif]

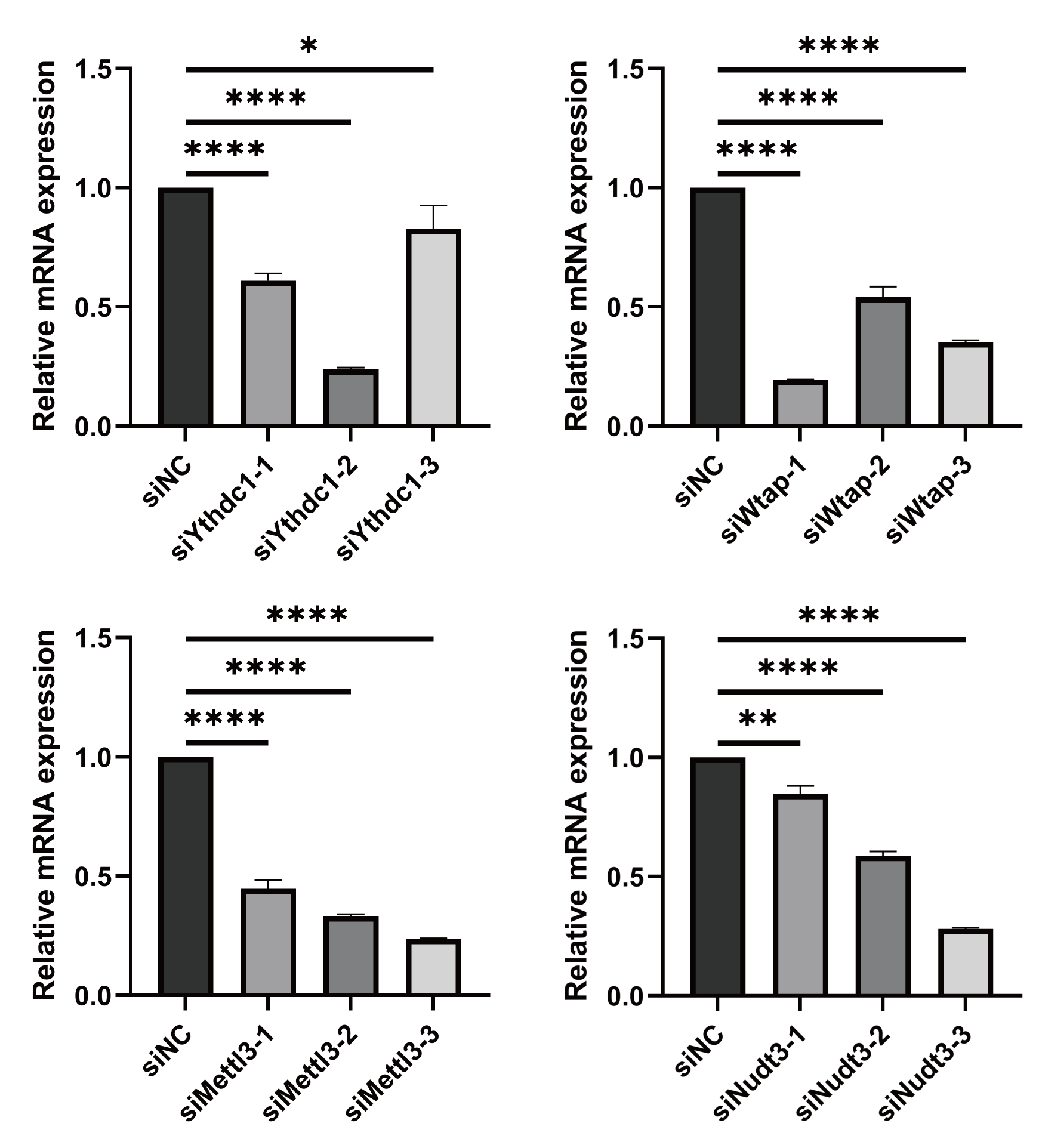

Supplement: Supplementary file 7 — Additional file 7: Fig. S4. Efficiency verification of RT-qPCR for siRNAs targeting core DEMRGs. The P-values were shown as: *P < 0.05, **P < 0.01, ****P < 0.0001. [file 40001_2024_1928_MOESM7_ESM.tif]

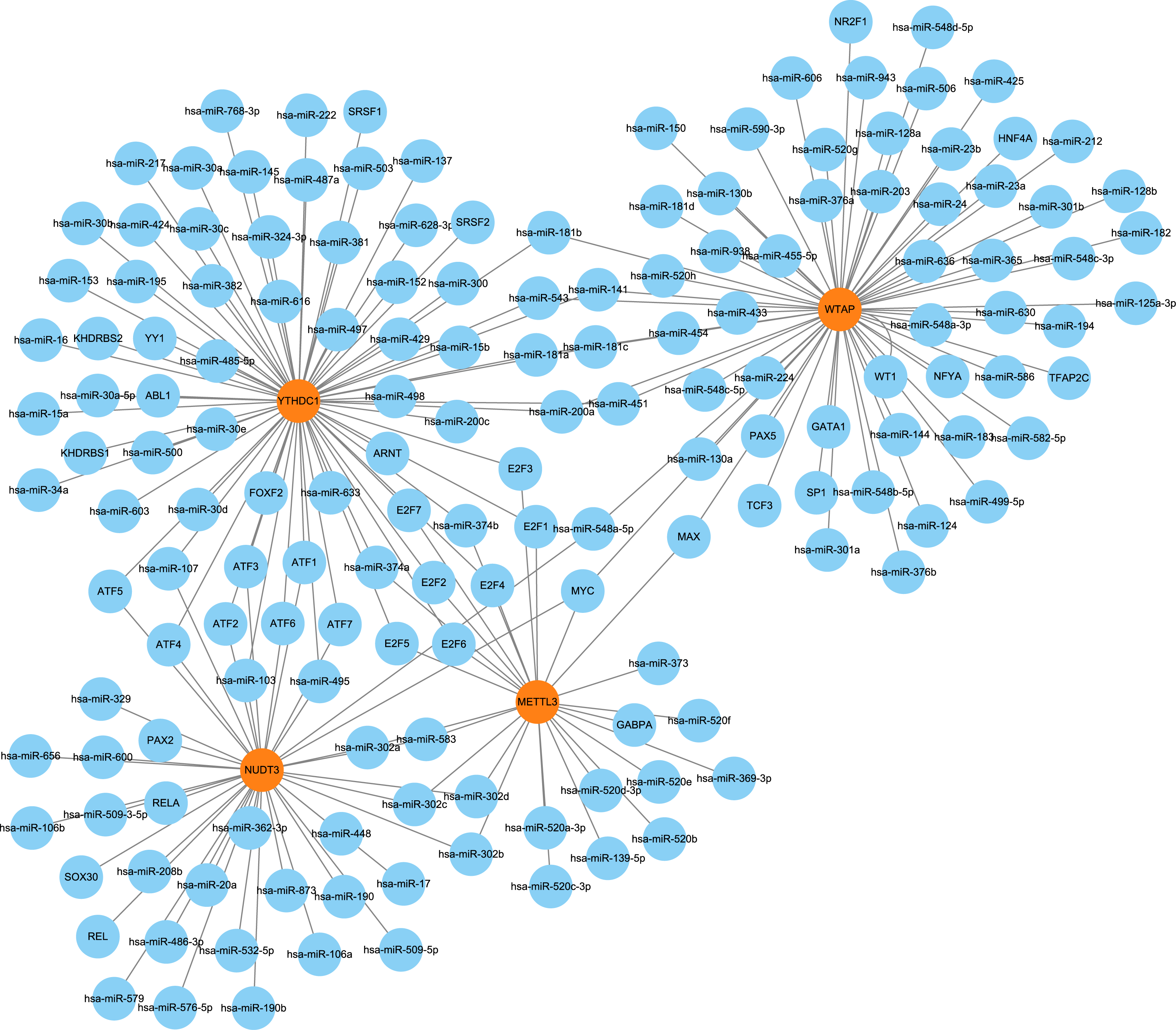

Supplement: Supplementary file 8 — Additional file 8: Fig. S5. Construction of a network consisting of the potential upstream miRNAs and transcription factors of core DEMRGs. [file 40001_2024_1928_MOESM8_ESM.tif]
